# The XVIII Brazilian Congress of Assisted Reproduction

**DOI:** 10.5935/1518-0557.20140009

**Published:** 2014

**Authors:** Joaquim Roberto Costa Lopes

**Affiliations:** President of the XVIII CBRA

Since Salvador was chosen to host the XVlll Brazilian Congress of Assisted Reproduction in August 2012, a multi-professional team took on this challenge with the commitment to perform an event that can mark the history in the SBRA (Brazilian Society for Assisted Reproduction) congresses universe.

The difficulties in obtaining public resources in which makes the congress aim a better medical assistance, made us adjust to the budget provided by the sponsor complemented by the registrations of the professionals who work in the area. This made us implement a management program based on reality by certifying we offer a congress rich in information and full of harmony moments.

The total was 24 months marked by the “thinking and breathing” addressing to offer an up-to-date and a scientific schedule focusing on daily subjects and its specialties. Besides controversial topics that deserve to be highlighted since they are debated with different points of view, we will also have conferences, round tables, study courses and panels. In this masterful stage there will be thirteen international professors and almost one hundred Brazilian presenters highly qualified.

We chose to host the event at the Gran Hotel Stella Maris Resort & Convention which is a complex located in one of the most lovely beaches in Salvador and only 7 kilometers away from the Salvador airport.

All of this effort aimed not only to create a scientific and diversified schedule to the experts but also to extend to the gynecologists who are interested in the reproduction topics. The members of the congress will also be able to witness magical moments of social activities, which will provide the opportunity to gather with old friends.

You will find the enchantment of Bahia along with the happiness of its song and a very special culinary. Be welcome! Then you will finally figure out the famous Brazilian expression “what the Baiana has…”

**Joaquim Roberto Costa Lopes**

President of the XVIII CBRA

